# Cognitive Deficits: Verbal and Semantic Fluency in People Living with HIV and AIDS

**DOI:** 10.2174/1570162X21666230613124240

**Published:** 2023-09-05

**Authors:** Ganka Ivanova, Rakan Alhrahsheh, Kaloyan Kukov

**Affiliations:** 1 Applied Psychology Department, Al Ain University, Abu Dhabi, UAE;; 2 Applied Sociology Department, Al Ain University, Abu Dhabi, UAE;; 3 Clinical Psychology Department, Medical University, Sofia, Bulgaria

**Keywords:** Asymptomatic neurocognitive disorder, cognitive deficiency, HIV and personality, language deficit, verbal and semantic fluency, neurocognitive

## Abstract

**Background:**

Since the beginning of the HIV epidemic, the virus has taken millions of lives worldwide. The United Nations AIDS Fund’s statistics reported that deaths caused by HIV-related conditions and AIDS were about 39 million from the beginning of the epidemic to 2015. The united global efforts to fight the virus are considerably changing the indicators, such as mortality and morbidity, but the challenges remain. The total number of people living with HIV in Bulgaria as of 12^th^ May, 2015, was 2,121. As of 30^th^ November, 2016, the official data reported 2 460 people living with HIV. As of 13^th^ February, 2017, 2 487 individuals were HIV-seropositive. Approximately 60% of people with HIV are prone to developing cognitive impairment due to the infection.

**Objective:**

This study aimed to know the level of cognitive deficiency, in particular, the verbal and semantic fluency of people living with HIV and AIDS.

**Methods:**

In this study, a comparative analysis was carried out. The Stewart test was used to compare the average independent samples. For clarity, the average values, the test statistics, and the estimated significance levels are presented in the tables. Additionally, a statistical mechanism of factor selection was used by the forward stepwise method. The Wilks' Lambda statistic reported values between 0 and 1, with values close to zero indicating good discrimination of the model.

**Results:**

According to this research, the HIV positives participants generated fewer verbs than the ones from the control group. The data were partially confirmed by the present study. There were differences in terms of both adjectives and nouns among people living with HIV and AIDS.

**Conclusion:**

The study data proves that language deficits are detectable in neurocognitive testing of HIV. The overall hypothesis of the study has been confirmed. The language impairments are primarily qualitative and can be used as a marker for the initial and subsequent therapy assessment.

## 1 INTRODUCTION

Language deficits in people living with HIV and AIDS are part of the neuropsychological disorders accompanying conditions with different severity, such as asymptomatic neurocognitive disorder, mild motor neurocognitive disorder, and HIV-associated dementia. This study presented the research findings on the neurocognitive deficits in the phase of the asymptomatic neurocognitive disorder.

This research aims to assess language impairment (verbal and semantic fluency) in people with HIV and AIDS. For this purpose, a total of 34 men and women living with HIV and AIDS (PLWHA), aged between 18 and 75, and 34 HIV- negative participants were examined. The clinical sample was collected in an HIV and AIDS treatment ward, where patients signed an informed consent as part of their medical history. The control group, the HIV-negative participants, agreed to participate in the anonymous research. The research procedure included verbal and semantic fluency tests (IST, COWAT), statistical analysis for specificity and sensitivity, discriminant, multifactorial analysis of variance, cluster analysis, and t-tests. The data from the research showed that the verbal and semantic fluency tests were sensitive but did not have a high discriminative function. It is further observed that people living with HIV and AIDS suffer from impairment in language and semantic relationships.

### 1.1 Research Questions

The following research questions were sought to be answered in this study:

RQ1. Are there statistically significant differences between people's cognitive deficits linked to their education levels and family status?

RQ2. Are there statistically significant differences between the semantic fluency of people linked to their level of education and family status?

RQ3. Are there statistically significant differences concerning verbal fluency between men and women in the HIV-positive groups?

### 1.2 Hypotheses

Hypothesis 1: There are statistically significant differences at the significance level (*p =* 0.05) regarding cognitive deficits among people in the HIV positive related to their level of education and family status.

Hypothesis 2: There are statistically significant differences at the significance level (*p =* 0.05) regarding semantic fluency among people with HIV positive related to their level of education and family status.

Hypothesis 3: There are statistically significant differences at the significance level (*p =* 0.05) between men and women in the HIV-positive groups concerning verbal fluency and cognitive deficits.

### 1.3 Theoretical Framework

The language function, a part of the global assessment of neuro-cognitive deficits, is related to the assessment of the ability to name verbal and semantic fluency.

Evidence of language deficits can be seen in HIV-infected children [1]. It is considered that there is evidence of subtle speech and language impairment in adults. Aphasia is rarely discovered in HIV-infected patients. It occurs when opportunistic infection develops (*e.g*., progressive multifocal leukoencephalopathy), resulting in a perisylvian lesion [2].

Villanueva *et al.* [3] found evidence of mild motor speech disorders in six HIV patients, which he defined as ataxic dysarthria. Other research reported subtle vocal abnormalities in HIV cases [4], such as decreased speech volume, shorter phonation, and mild vocal tremor [5]. However, it is assumed that receptive and expressive speech is without disorders in individuals with HIV [6], but if there are any mild impairments, these may also be present in the asymptomatic phase. Consequently, they worsen the condition [7]. Despite these difficulties, the formal assessment of the nominative function of the speech (animal enumeration) is within the norm [8], but research has shown individual differences [9]. Some mild impairments in the pragmatic aspects of communication, such as inappropriate conversation interruptions with pauses, poor vocabulary choices, and reduced fluency, can be observed in individuals with HIV [6]. Verbal fluency disorders are the most commonly identified speech deficits. They are found in about 40% of people with HIV. The deficits in verbal fluency may be mild, but with the progression of the disease, they increase to moderate in people with advanced HIV [10]. Some meta-analyses reported significant effects for categories associated with assigning a particular letter and category [10], which is further related to the state of the frontal systems, similar to the frontal lobe lesions [11]. The research on HIV-associated verbal fluency disorder is associated with the neurodegeneration of the frontostriatal system, including atrophy of the caudate nucleus [12].

At the level of the cognitive processes, deficits in verbal fluency can arise due to several factors, such as bradyphrenia, degradation of the stored semantic network, and/or gaps in switching between the lexico-semantic categories during word retrieval [13]. There are several research works [14] that model sequential word generation within a lexical-semantic category and switching (*i.e*., detachment from one cluster and then identification and access to another category from which to generate words) to evaluate the hypothesis that the HIV-associated verbal fluency impairment may be a function of the executive switching dysfunction, *i.e*., secondary to the frontostriatal neurotoxicity. According to Woods [15], HIV-associated verbal fluency impairment is associated with a specific impairment in the search and retrieval process from the lexical-semantic network (91% of the errors are in the context of phonemic switching). Millikin [16] reported that the deficits in categorial and letter fluency are due to switching rather than grouping. According to Ludicello *et al*. [17], impairments in categorial fluency are observed in immunocompromised HIV-infected individuals. The categorial deficits correlate with the executive functions, the working, and the semantic memory. These studies suggested that the HIV-associated deficits in verbal fluency may be due to the difficulties in switching between the semantic memory network (bradyphrenia or semantic memory impairment). They can be a function of executive dysfunction. The goal-directed search and retrieval processes are associated with the dysregulation of the frontotemporal systems. These semantic memory patterns also suggest that disturbances in the frontotemporal network can exist.

Perhaps due to the lack of severity, the HIV-associated deficits in verbal fluency are unreliable predictors of impaired daily functioning. More effective [18] (Woods, 2005a) are some new studies using verbs [19]. They demonstrated greater impairment in individuals with HIV, a better predictor of impaired daily functioning in people with HIV [20] compared to the standard tests of fluency measures, such as the FAS and IST.

### 1.4 Verbal and Semantic Fluency Tests

Fluency, *i.e*., the rapid generation of something, was developed in accordance with the conceptual framework of the neural circuits. The generation of nouns, in particular, is associated with the temporoparietal circuits and the semantic memory network, whereas the generation of verbs is associated with the frontal systems, executive functions, and motor planning [21].

### 1.5 Phonemic Fluency (COWAT)

For this version, the subject has to orally reproduce as many words as possible, beginning with a certain letter for a certain amount of time, usually 1 minute. As Marshall [22] emphasized, “word fluency” is misleading because verbal productivity in a conversation or a composition of long sentences is not assessed. Instead, the test measures the reproduction of individual words over a specified period and under specified conditions (*e.g*., a particular letter of the alphabet). Thus, to avoid confusion with the dimensions of speech fluency/nonfluency, Benton [23] coined the term Controlled Oral Word Association (COWA). However, the test is well known by the common name “verbal fluency.”

For Bulgaria, the letters К, (K) П, (P)С(S) are chosen. After consultations with Prof. Sv. Kostova (Bulgarian Academy of Science, Institute of Computational Linguistics), the choice of the following letters was made due to several reasons.

The letters with which start the largest number of words within the Bulgarian Wordnet are mentioned in Table **1**.

In Bulgaria, the standardized test is Isaac’s test, which distinguishes a healthy person from a patient with mild dementia. The threshold is 27/28, with 100% sensitivity and specificity for people under 65 years and 94% sensitivity and 90.14% specificity for patients over 65 years.

The available data coincide with the proposed critical values from the nine-year epidemiological research PAQUID [24], which identifies patients at high risk of developing dementia nine years before developing the clinical dementia symptoms. When the group of patients with mild cognitive impairment was expanded, the cut-off values in our study increased to 30/31 with a sensitivity of 91.14% and specificity of 71.05% for those under 65 years and a sensitivity of 82% and specificity of 73.2% for those over 65 years. Similar rates were reported by Amieva and co-authors in the 20-year epidemiological research PAQUID [25], where Isaac's test was used as one of the predictors of increased risk of dementia. The values found in the range of 30/31 are risky for patients over 65 years of age and who received an education of 17 years or more [26].

## 2 MATERIALS AND METHODS

### 2.1 Data from Empirical Research

The research included 68 men and women, 34 men and women living with HIV and AIDS (PLWHA), and 34 HIV-negative participants aged 18 to 65. The initial intention was to include 100 participants in total; however, 32 (16 living with HIV and 16 not living with HIV) refused to participate in the study. In terms of education, the research included people with primary, secondary, and university education, married and single, with a viral load below and above twenty copies in 1 ml of blood. With a load below 20 copies, a person is considered to be in remission of the infection. If the CD4 cells are below and above 350, it indicates the degree of impairment/recovery of the examined person. If there are below 350 cells, maintenance therapy is necessary (the measurement of the number of CD4 cells indicates the recovery and remission phase) and the presence or absence of co-infection (hepatitis B).

### 2.2 Demographic Parameters in the HIV/AIDS Group

The average age of the group was 41 years and nine months, the minimum age was 25 years, and the maximum was 65 years. On average, the participants in the study group had 583.12 CD4 cells, the minimum being 116 CD4 cells and the maximum being 1300 CD4 cells. The viral load ranged from less than 20 copies to 310 000 copies, with an average of 29 382.73. The years since the detection of the virus varied from 1 to 27 years, with an average of eight years and three months.

The participants included 21 men (60.6%), 13 women (39.4%), 4 people with primary education (12.1%), 20 people with secondary education (57.6%), and 10 people with university education (30.3%). There were one man and three women with primary education, 14 men and six women with secondary education, and five men and five women with university education: 26 people with a viral load below 20 copies (75.8%) and 8 people with a viral load above 20 copies (24.2%) (Table **2**).

### 2.3 Demographic Parameters of HIV-negative Participants

The average age of the group was 39.8 years, with a standard deviation of 15.2 years. The HIV-negative group included 10 women (29.4%) and 24 men (70.6%), 5 people with primary education (14.7%), 18 with secondary education (52.9%), and 11 with university education (32.4%) (Table **3**).

### 2.4 Statistical Analysis

The statistical significance of the results from the research (effects) is shown by the value of the estimated significance level of the study, which is the *p*-value. The *p*-value is interpreted as the probability of error in rejecting the null effect hypothesis (first-order error). The smaller the p, the more certain it is to reject the null hypothesis and accept the assumption that the observed effect is statistically significant. The null hypotheses are usually rejected at *p <* 0.05 (<5%). At values close to the threshold, the effect is marginally significant.

In the comparative analysis, the Stewart test was used to compare the average independent samples. For clarity, the average values, the test statistics, and the estimated significance levels are presented in the Tables. The discriminant analysis established the separating power of a group of factors on a nominal dependent variable. Additionally, statistical methods of factor selection was used by the forward stepwise method. The Wilks' Lambda statistic reported values between 0 and 1, with values close to zero indicating good discrimination of the model. The partial Lambda statistics demonstrated the individual contribution of each variable.

The cluster analysis groups the objects by similarity, considering the agglomeration levels.

The sensitivity is defined as the probability of a correct positive assessment regarding the subject's affiliation (within this research) with the risk group.

The analysis of variance is a statistical method for analyzing the dependencies, where the resulting phenomenon is represented at a strong scale, and the factors can be categorical variables. As a procedure, the analysis of variance is a statistical hypothesis testing procedure in which Fisher's F-criterion is used. In this study, SPSS-18 was used for the statistical processing of the data.

### 2.5 Language Function Tests

#### 2.5.1 Phoneme Fluency (COWAT)

For this version, the examinee was asked to orally reproduce as many words as possible, beginning with a particular letter, for a specified amount of time, usually 1 minute. As Marshall [23] highlighted, “word fluency” is misleading because verbal productivity in a conversation or the composition of long sentences is not assessed. Instead, the test measures the reproduction of the individual words over a specified period and under specified conditions (*e.g*., a given letter of the alphabet). Thus, to avoid confusion with the dimensions of speech fluency/nonfluency, Ruff *et al*. [27] preferred the term Controlled Oral Word Association (COWA). However, the test is well known by the common name “verbal fluency.”

The letters К (K), П(P), С(S) were chosen for Bulgaria after correspondence with Prof. Svetla Kostova from the Bulgarian Academy of Sciences, Institute of Computational Linguistics. These letters were selected on the basis of an analysis of the letters with which begin the largest number of words in the Bulgarian Wordnet. Each participant had to list, within 1 minute, words starting with the letters К, П, and С, excluding individual and city names.

#### 2.5.2 Semantic Fluency (ISAC Set Test)

The ISAC test [28] assessed verbal fluency, the retrieval from semantic memory under a significant executive load. It requires the generation of words from specified semantic categories for 15 seconds. In this study, participants were asked to make a list of fruit, colors, cities, and animals; each task was performed in 15 seconds.

The pilot study was conducted individually from November 2012 to December 2018 in the HIV/AIDS treatment ward of the infectious diseases clinic at the Multiprofile Hospital for Active Treatment St. Marina, Varna.

## 3 RESULTS

### 3.1 Data from Independent Research Related to Sensitivity and Specificity

Data from independent research related to sensitivity and specificity are: In the IST test, for fruits, 0.707 sensitivity was reported, for colors, 0.626 sensitivity, and for cities, low 0.476 sensitivity. Categorical fluency (animals) showed 0.740 sensitivity.

### 3.2 COWA

**The letter K**: the subtest shows 0.662 sensitivity. **The letter П**: the subtest shows a sensitivity of 0.502.

**The letter C**: the subtest shows 0.493 sensitivity.

### 3.3 Discriminant Analysis of the Isaac Set Test

#### 3.3.1 Discriminant Analysis of the Isaac Set Test: Fruit

The values of the total Wilks' Lambda coefficient=0.835 and total Sig coefficient=0.000 <0.12 show no statistically significant discriminant ability for the subtest.

#### 3.3.2 Discriminant Analysis of the Isaac Set Test: Colors

The total Wilks' lambda coefficient values =0.945 and total Sig coefficient =0.000 <0.22 show no statistically significant discriminant ability for the subtest.

#### 3.3.3 Discriminant Analysis of the Isaac Set Test: Cities

The values of the total Wilks' lambda coefficient=0.991 and total Sig coefficient=0.000 <0.63 show no statistically significant discriminant abilities for the subtest.

#### 3.3.4 Discriminant Analysis of the Isaac Set Test: Animals

The values of the total Wilks' lambda coefficient=0.968 and total Sig coefficient=0.000 <0.35 show no statistically significant discriminant ability for the subtest.

#### 3.3.5 Discriminant Analysis of Category Animals

The values of the total Wilks' lambda coefficient are determined by the given table: Wilks' lambda=0.968 and Sig=0.000 >0.14, which means that there are no statistically significant discriminant abilities for the test.

The above classification table shows probabilities; the probability of not being in the group of “people living with HIV and AIDS” and not falling into this group in the analysis is 50.0%. The likelihood of not being in the group of “people living with HIV and AIDS” but falling into this group is 50.0%

### 3.4 Discriminant Analysis of the Controlled Oral Word Association Test

#### 3.4.1 Discriminant Analysis of the COWA-K Test

The Wilks' lambda coefficient=0.936 and the Sig coefficient=0.000 >0.18 show no statistically significant discriminant abilities for the subtest.

#### 3.4.2 Discriminant Analysis of the COWA- П Test

The Wilks' lambda coefficient=0.990 and the Sig coefficient=0.000 >0.61 show no statistically significant discriminant abilities for the subtest.

#### 3.4.3 Discriminant Analysis of the COWA - C Test

The Wilks' lambda coefficient=1.000 and the Sig coefficient=0.000 >0.92 show no statistically significant discriminant ability for the subtest.

For the IST, animals and COWA tests were performed by independent samples t-tests to be determined if there was a difference between the people living with HIV and AIDS and the group of healthy people.

In examining the fluency with IST, there are two differences in relation to the subtest: fruit per 15sec, where the people with HIV and AIDS have a significantly (t(27) = 2.32, *p <* 0.05) lower score (M=7.07, SD=1.34) than the healthy people (M=8.33, SD=1.59). The other difference is in the subcategorical fluency enumeration of animals for 1 min., the people with HIV and AIDS have a significantly (t(27) = 2.62, *p <* 0.05) lower score (M=11.18, SD=3.14) than the healthy people (M=8.26, SD=3.03).

The results for the people with HIV and AIDS, listed by category words with the letter П, show that the people living with HIV and AIDS have a significantly (t(27) = 2.51, *p <* 0.05) lower score (M=10.93, SD=5.07) than the healthy people (M=10.07, SD=3.88).

The other difference is in the subcategorical fluency enumeration of animals for 1 min.

For the people with HIV and AIDS, the enumeration by category of domestic animals has a significantly (t(24) = 2.58, *p <* 0.05) lower score (M=16.38, SD=6.24) than the healthy people (M=20.62, SD=1.60).

For the people with HIV and AIDS, the enumeration by category of wild domestic animals has a significantly (t(24) = 3.48, *p <* 0.05) lower score (M=16.46, SD=6.33) than the healthy people (M=20.15, SD=2.82).

For the people with HIV and AIDS, the enumeration by category of African animals has a significantly (t(24) = 2.47, *p <* 0.05) lower score (M=12.62, SD=6.80) than healthy people (M=19.32, SD=2.25) [29,30]. Meta-analysis demonstrated a difference, albeit small, in the verbal and semantic fluency between HIV-negative and HIV-positive people. The data confirmed that this deficit is associated with the executive processes.

For semantic fluency, an analysis was performed featuring the differences between the two groups, those with HIV and AIDS and those with HIV negative. The words beginning with К, П, and С were divided into nouns, verbs, and adjectives. For the individuals with HIV and AIDS, the listing by category of adjectives with K has a significantly (t(24) = 1.99, *p <* 0.05) lower score (M=1.08, SD=1.44) than one of the healthy individuals (M=4.23, SD=5.51).

For the people with HIV and AIDS, the enumeration by category of nouns with C has a significantly (t(24) = 2.18, *p <* 0.05) lower score (M=7.85, SD=2.85) compared to the healthy people (M=10.62, SD=3.57).

For the people with HIV and AIDS, the enumeration by category of nouns with П has a significantly (t(24) = 3.03, *p <* 0.05) lower score (M=7.77, SD=2.71) than the healthy people (M=11.42, SD=3.45).

For the people with HIV and AIDS, the enumeration by category of verbs with П has a significantly (t(24) =,23, *p <* 0.05) lower score (M=0.92, SD=0.60) than the healthy people (M=3.85, SD=1.93).

**Fig. (1):**
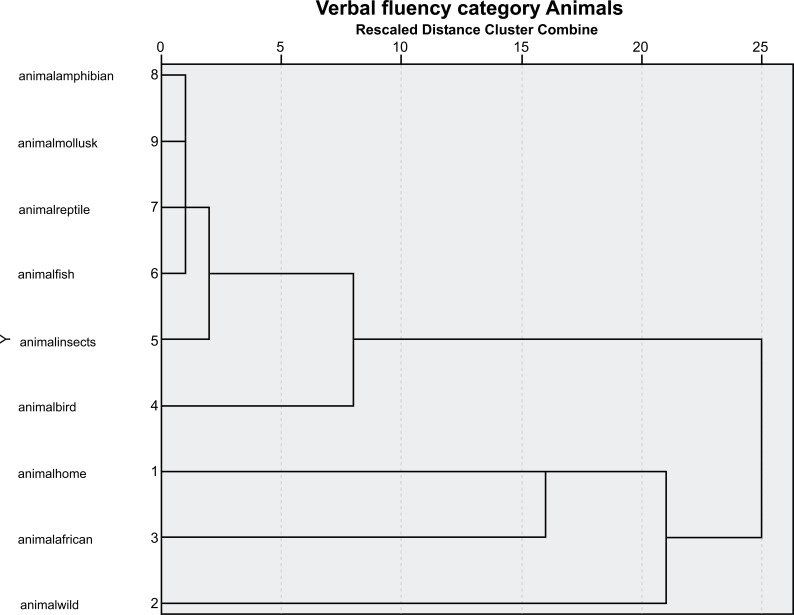
Dendrogram verbal fluency group of people living with HIV and AIDS.

**Fig. (2):**
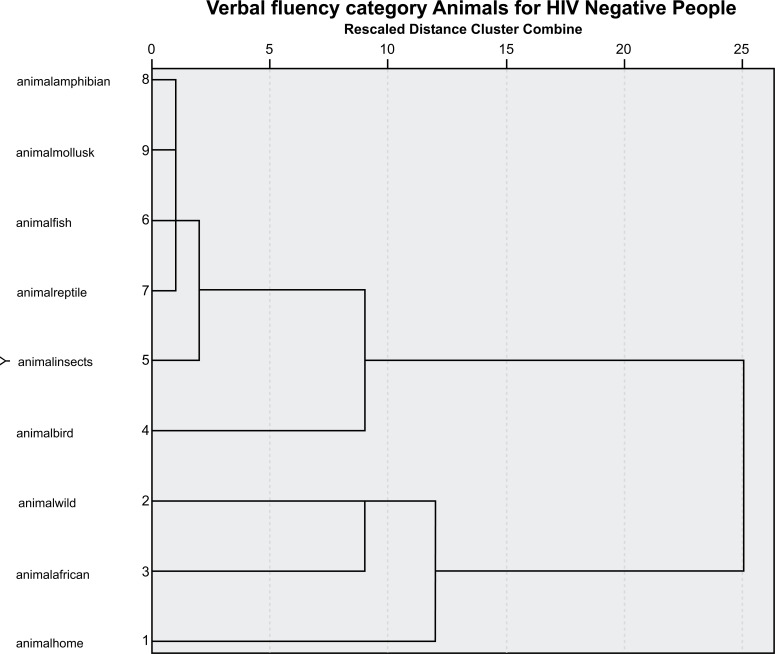
Graph of the verbal fluency group of HIV-negative participants (control group). Dendogram verbal fluency of HIV-negative participants.

**Fig. (3):**
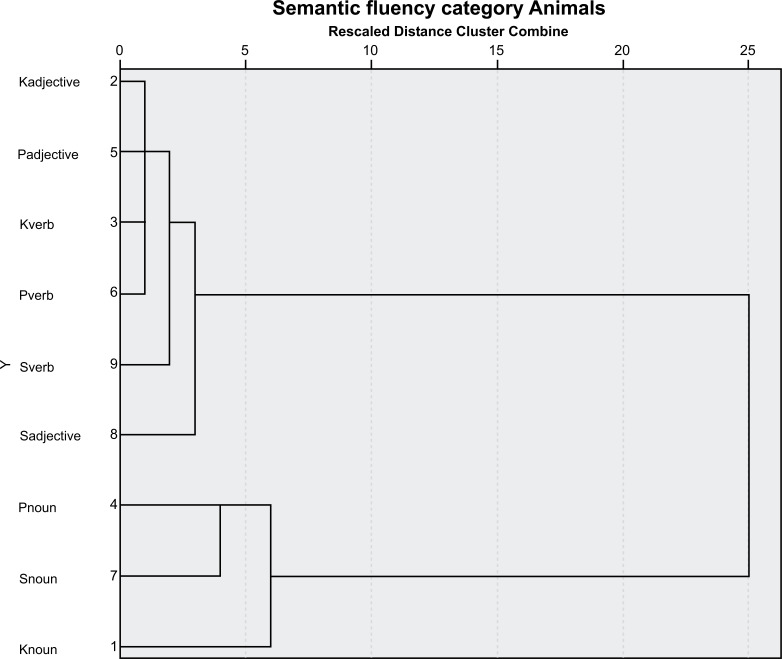
Semantic fluency of the people living with HIV and AIDS. Dendogram 3. Semantic fluency of the people living with HIV and AIDS. K (K) adjective, П (P) adjective, K (K) verb, П (P) verb, C (S) verb, C (S) adjective, П(P) noun, C(S) noun, and K(K) noun There are two clusters observed in the analysis: one is composed of K (K) adjectives, П (P) adjectives, K(K) verbs, П(P) verbs. C(S) verbs, and C (S) adjectives. The second cluster is composed of П(P) nouns, C(S) nouns, and K(K) nouns. Dendrogram semantic fluency of HIV-negative participants (control group).

**Fig. (4):**
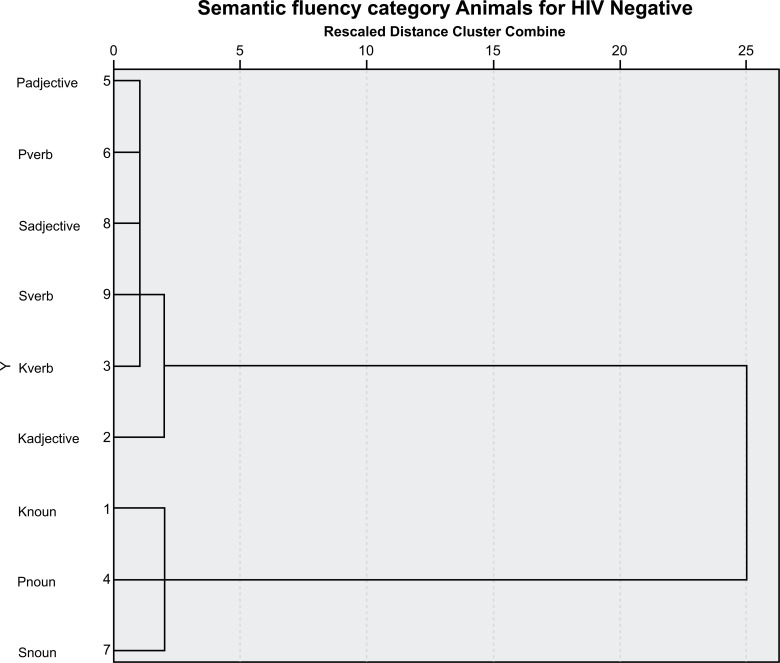
Dendrogram semantic fluency of HIV-negative participants (control group). П (P) adjective, П (P) verb, C(S) adjective, C (S) verb, K(K) verb, K(K) adjective, K(K) noun, and П(P) noun In the analysis, an association was observed between the П adjectives, П verbs, C adjectives, C verbs, K verbs, and K adjectives, in addition to K, П, and C nouns. As can be seen, a category shift occurs between the two groups. For the IST (fruit, colors, cities, animals), the effect of these variables was not significant (F=3.23, *p*>0.05). The effect was close to being significant because of the way education affected IST. For the enumeration of animals for one minute, the effect of these variables was not significant (F=0.79, *p*>0.05). For COWA, the effect of these variables was not significant (F=1.52, *p*>0.05).

A cluster analysis featuring the data from the research is presented in the graphs below (Figs. **1**-**4**).

The analysis demonstrated that for the group of people living with HIV and AIDS, the closest categories are amphibians, mollusca, reptiles, fish, and insects. In addition, birds and a separate cluster of domestic, African, and wild animals are also included in this category.

The analysis demonstrated that for the group of HIV-negative participants, the closest categories are amphibians, mollusca, reptiles, fish, and insects. Added to these are birds, and in a separate cluster, wild, African, and domestic animals are also included in this category. As can be observed from the two graphs, the proximity of animal species changes for the people living with HIV and AIDS.

A series of independent samples t-tests were also performed to determine the difference between the women and men in the HIV-positive group concerning the IST and COWA tests. No significant differences were observed.

For the IST, COWA tests were performed by independent samples t-tests to determine the difference between the people in the presence of a viral load below and above 20 copies in the HIV-positive group. No significant differences were found for the IST, animals, or COWA tests.

Similar tests were performed for the CD4 cell parameter below and above 350 in the HIV-positive group. No significant differences were observed. Similarly, no significant differences were found for IST, animals, COWA tests, and their errors.

There was also no significant difference in whether HIV-positive participants received therapy. The multivariate analysis of variance for the education, as a fixed factor, and the IST, animals, and COWA tests, as dependent variables, showed no significant (F (2, 12) = 1.61, *p =* 0.18) differences in the level of education of the HIV positive group, as well as in terms of the family status as a fixed factor. The IST, animals, and COWA tests, as dependent variables, showed no significant (F (2, 12) = 0.93, *p =* 0.53) differences in the family status of the HIV-positive group.

## 4 DISCUSSION

The general hypothesis of the research is related to the assumption that there will be differences between the two groups of people living with HIV and AIDS and one of the HIV-negative participants in terms of verbal and semantic fluency. It is assumed that differences will be observed in demographic parameters, such as gender and education and medical parameters, such as viral load below and above 20 copies and CD4 cell level.

To understand language disorders, one must understand the concept of semantic and verbal fluency [29]. Fluency is the ability to extract information from a semantic chain. According to Almazova *et al.* [30], semantic networks aim to explain how individual meanings and senses connect into a common system. Almost all models of this kind postulate the existence of semantic nodes and connections between them. The hierarchical network attempts to map the relationships between the semantic units and the features that characterize each of them. In such a network, there is a relationship of subordination between the semantic nodes. In the higher category semantic node “animals,” there are multiple subordinate semantic nodes, *e.g*., “birds” and “fish,” which, in turn, have specific representatives depicted in lower-lying subordinate semantic nodes. For example, in “birds,” subordinates can be found “canary” and “ostrich.” Each of these semantic nodes has its distinctive properties. The model becomes the basis for research on the semantic distance between the words that use the reaction time for judgments, such as “Canary is an animal” and “Canary is a bird.” If the model is consistent with reality, the verification of the first statement will take longer than the verification of the second because to establish if the first statement is accurate, we need to consider two semantic nodes that intersect while establishing if the second one is true, we have only one. An effect related to the category size was found, *i.e*., the more members it has, the longer the search takes. For the Bulgarian language, detailed research on the semantic distance was presented by Gerganov [31]. The hierarchical network model was later revised. The hierarchy was dropped and replaced by massive interconnectivity. According to Marshall [22], the data for the category of “Animal” generated expected results; the first associated animal is the lion, which is historically and culturally conditioned for the country. After that, the animals were sorted into the domestic and wild animal categories. The distribution of activation is considered to be a basic principle. In this form, the model assumes that activating a lexical unit facilitates the recognition of an association with the unit words, similar to an electrical impulse running between the connected objects. As in the previous version, the semantic units are represented as nodes, but the nodes can now also establish properties; “cherry” is associated with “red,” and “red” with “blue,” “orange” and “green.” The distance between the semantic units reflects the strength of the relationship between them. “Canary” is more strongly related to “sing” and less strongly to “skin.” This is how the model reflects the modern tendencies of connectionism in understanding information processing. The distribution of activation is automatic, and the speaker cannot control it. It differs from top-down processing, where the broader meaning context affects the interpretation. One directs the rapid fading of images because doing so prevents the simultaneous activation of too many units. The effects of the distribution of activation demonstrate the semantic relationship of different types. Moreover, the strength of the effect depends on the strength of the association: “dog” and “cat” are in the same class as “pig” and “horse,” but the relationship between the former two is much stronger because of their more frequent co-occurrence. There is undoubtedly a relationship between the function, the language, and the substrate, the brain.

It has been suggested that the generation of verbs is mainly associated with the circuits, which connect the frontal lobes with the basal ganglia, whereas the generation of nouns is primarily mediated by the dominant temporo-parietal networks on the left. As per this hypothesis, it is suggested that the verbal fluency associated with generating verbs indicates greater sensitivity to the pathophysiological changes in the frontal-basal ganglia (*e.g*., dementia in Parkinson's disease). Woods’s study [29] reported that significantly fewer verbs were generated in the HIV-positive group. According to this research, the HIV positives participants generated fewer verbs than the ones from the control group. The data were partially confirmed by the present study. There are differences in terms of both adjectives and nouns among people living with HIV and AIDS.

The relationship between language and education is highlighted. According to research by Mathuranath *et al.* [32, 33], the level of education affected verbal fluency. This study included healthy adults. They were examined with the categories of animals and letters. The data obtained did not confirm, in a definite way, that education is a factor in the word's generation of healthy adults. A meta-analysis study by Ludicello *et al*. [10] reported that the effect of the education factor in the HIV-positive group was small but still existed. In terms of gender, the data obtained showed that gender is not a significant factor, which is an expected result. HIV is not gender-differentiated. The data at hand supported the hypothesis that no gender differentiation is observed in the HIV-positive group; such data were observed in the research conducted by Iuducello [18].

Antiretroviral therapy is lifesaving, but there are other factors, such as education and individual resistance [34]. Moreover, no effect of the viral load on the neurocognitive impairment of HIV patients on monotherapy (n = 51) and combination antiretroviral therapy (HAART) (n = 90) was reported. Here, the hypothesis arises that, possibly due to the limitations of the viral load in 1 ml. of blood, the difference in the viral load in the cerebrospinal fluid may also have an effect. Letendre [35] reported that regardless of how the viral load was measured, whether, in blood or cerebrospinal fluid, no relationship was observed between the neurocognitive impairment and the viral load [36], but this research confirmed this association. Despite using markers, such as CD4 cells, to assess the treatment progress, the findings did not support the results of a study by Elkin *et al.* [37], which found poorer cognitive functioning at lower CD4 cell levels. This may be because, in the asymptomatic neurocognitive disorder, there are still compensatory mechanisms in the brain that affect cognition and verbal fluency in particular. The relationship between the immune system, cognition, and behavior has been known for a long time; these are called integrative mechanisms [36].

However, the findings of this study were not consistent with the results reported by Uchiyama *et al.* [38, 39], which reported no significant differences in the asymptomatic neurocognitive impairment phase.

Although no difference was observed in the language functions in the HIV-positive group, family support is an important part of the overall biopsychosocial service model. Even though the family environment does not directly affect language functions, it may indirectly have influences. The relationship between the family environment and the support for people living with HIV and AIDS may be a latent factor, similar to the emotion. Awareness of the risk of HIV infection is still poor [40, 41]. A phenomenon described as the separation of knowledge from behavior was detected. Misconceptions that affected behavioral patterns were identified as critical factors. Therefore, we suggest that cognitive behavioral therapy may alter the actual effect of AIDS prevention interventions [41]. Research demonstrated that without family support, individuals with HIV and AIDS have more negative emotional experiences on the anxious-depressive register [33], leading to a decline in overall cognitive functioning.

## CONCLUSION

This study proved that language deficits are important to neurocognitive testing in HIV. The overall hypothesis of the study has been confirmed. The language impairments are primarily qualitative and can be used as a marker for the initial and subsequent therapy assessment. The performed tests were brief; they did not take much of the clinicians' time, and they were not difficult to process, and due to these circumstances, they did not require extensive training.

The limitations of the research are related to the small sample, which the pronounced stigma can explain toward this group: the people living with HIV and AIDS. Future research needs to assess the functional aspect, *i.e*., the language, and the biological aspect, *i.e*., the cortex, as this would provide additional material for analysis. The research generated several hypotheses, such as the relationship between cognition and, in particular, the language functions with emotions, such as depression and anxiety. The results suggested that the answers given by the research are future questions requiring further research on the people living with HIV and AIDS.

## Figures and Tables

**Table 1:** Nouns, verbs, and all words within the bulgarian wordnets.

**Items**	**Symbols and Numbers**
NOUNS	П (P) 8329
	С (S) 7049
	К (K) 5647
	М (M) 3951
	Д (D) 3679
	А (A) 3618
	Б (B) 3357
	О (O) 3117
	Т (T) 2856
VERBS	П (P) 5327
	О (O) 3031
	И (E) 2565
	С (S) 2307
	З (Z) 1737
	Н (N) 1701
	Р (P) 1628
	В (V) 1345
	Д (D) 941
ALL WORDS	П (P) 26706
	С (S) 17971
	Н (N) 13319
	О (O) 12247
	К (K) 11155
	Р (R) 10348
	И (I) 9467
	Д(D) 8593
	В(V) 8392
	Б (B) 7831

**Note:** In the official spelling dictionary, most words begin with the letters: П (P), С(S), К(K), Н(N), М(M), Т (T), О(O), and Д.(D).

**Table 2:** Distribution of parameters in the HIV/AIDS group.

**Variable**		**Frequency**	**Percent**
Gender	Female	21	60.6
	Male	13	39.4
	Total	34	100
Education	Primary	4	12.1
	Secondary	20	57.6
	University	10	30.3
	Total	34	100
Age	Less than 18	3	9.00
	18-30	5	15.0
	31-40	4	12.0
	41-50	10	30
	50-60	6	17
	More than 60	6	17
	Total	34	100

**Table 3:** Distribution parameters of HIV-negative participants.

**Variable**		**Frequency**	**Percent**
Gender	Female	10	29.4
	Male	24	70.4
	Total	34	100
Education	Primary	5	14.7
	Secondary	18	52.9
	University	11	32.4
	Total	34	100
Age	Less than 18	4	11.0
	18-30	5	12.0
	31-40	11	31.0
	41-50	7	20.0
	50-60	5	15.0
	More than 60	4	11.0
	Total	34	100

## Data Availability

The data and supportive information are available within the article.

## LIST OF ABBREVIATIONS

AIDS: Acquired Immune Deficiency Syndrome
COWA: Controlled Oral Word Association
HIV: Human Immunodeficiency Virus
